# Coping strategies for handling stress and providing mental health in elite athletes: a systematic review

**DOI:** 10.3389/fspor.2023.1265783

**Published:** 2023-11-16

**Authors:** Barbara Nuetzel

**Affiliations:** Department of Psychology, Deutsche Hochschule für Prävention und Gesundheitmanagement (DHfPG), Saarbrücken, Germany

**Keywords:** elite athletes, coping strategies, stress, effects, mental health

## Abstract

**Background:**

The combination of stressors unique to the athletic context plus the sensitive developmental phase that elite athletes go through during their peak performance may increase the athletes’ vulnerability to mental health decrements. To emphasize the necessity to raise elite athletes' awareness of health risks, it seems to be essential to teach them coping skills to handle stress and to make affected athletes aware of how to recognize, evaluate, and articulate potential risks to their health.

**Objective:**

This systematic review analyzes coping strategies used by elite athletes to deal with stress and the effect of these strategies on mental health to identify gaps that future research could prioritize.

**Methods:**

The current review analyzes studies containing quantitative, qualitative, and mixed data and results, all of them focusing on coping strategies to deal with stress and the effect of coping strategies on elite athletes' mental health. Literature search for this systematic review took place between August and October 2023 and included the use of 3 electronic databases: PubMed, PsychINFO, SPORTdiscus.

**Results:**

There were initially 5,705 hits from 3 electronic databases, hand search and from a complementary search in Google Scholar. After the screening process and quality appraisal 30 studies were included. The analyzed study results point to a broad spectrum of coping categories, elite athletes make use of to deal with stressful situations. The results of this review underpin the necessity that especially young athletes being confronted with a wide range of stressors, need to be taught mental skills to cope with these stressors. In addition, teaching coaches and teammates about social support seemed to decrease elite athletes' stress reactions, such as anxiety or depressive symptoms.

**Conclusion:**

Coping in elite sporting settings is very complex and dynamic. There is evidence of coping being effective to buffer stress, but the interrelationships between stressor, appraisal of the stressor, application of a corresponding coping strategy and its effect especially in terms of mental health outcomes is still unclear because of lacking intervention-based study designs.

## 1. Introduction

For some time now, research on the impact of stress on mental health has been an increasingly important issue in elite sport (1).

The context of elite sports consists of various stressors (2), including competitive, organizational, and personal stressors, that have the potential to increase the risk of mental health related problems and mental illness in elite athletes (1). The stressors elite athletes experience during their competitive years may be of different nature. That means that interdependencies between stress and mental health related problems in elite sport, can vary from athlete to athlete (1). Previous approaches to stress or stress management clearly show that stress is one of the key health risk factors (3).

The combination of stressors unique to the athletic context plus the sensitive developmental phase (4) that elite athletes go through during their peak performance may increase the athletes' vulnerability to mental health decrements.

In this context, it seems important to understanding the age-performance-health relationship to promote the athletes' health (5). Significant for the onset and development of mental health-related problems in elite athletes is the concept of psychological strain which is characterized by a combination of perceived stress and the difficulty to cope with stress. If coping resources are extended beyond an elite athlete's capacity, stress-related symptoms of psychological strain may rise (6). To emphasize the necessity to raise elite athletes' awareness of health risks, it seems to be essential to teach them coping skills to handle stress and to make affected athletes aware of how to recognize, evaluate, and articulate potential risks to their health (7).

Since elite athletes are continuously confronted with the most varied challenges, environments, and stressors (2), it is not only from a biopsychosocial perspective (8, 9) to be desirable but also from the transactional stress perspective as well as from conservation of resources (COR) theory to develop flexible coping strategies against the potential negative impact of stressful life events (10, 11). The “classical” cognitive psychological approach of Lazarus (12) and Lazarus and Launier (13) sees the process of coping with stress as an equilibration process of an imbalanced psychophysical state. The question of coping effectiveness, however, is different in elite sports than in the field of clinical psychology. In contrast to people in everyday life, elite athletes are inevitably confronted with critical situations in competition.

To date, scientific attention has not explicitly focused to analyze the possibly effect of coping strategies to handle stress regarding mental health within the context of elite sports (14). Nicholls and Polman (15) reviewed the literature on coping in sport spanning 16 years (1988–2004) to examine evidence for the types of coping strategies applied by elite athletes and coping effectiveness, whereas Sarkar and Fletcher (16) focused on the interplay between stressors and protective factors and their influence on psychological resilience in elite athletes.

However, it is this knowledge of factors affecting athletes' mental health that is of tremendous importance to research. This is where the current systematic review comes in to contribute to this scenario aiming to analyze coping strategies used by elite athletes to deal with stress and the effect of these strategies on the mental health of elite athletes to identify gaps that future research could prioritize. As such the purposes to this systematic review are twofold:

To give an overview of the coping strategies elite athletes use to cope with stress (1) and to investigate the effectiveness of these strategies for coping with stress (2).

## 2. Methods

Methodologically, this systematic review is informed by the (PRISMA) guidelines (17, 18) that serve the generation of systematic reviews and meta-analyses. The PRISMA explanation was used as a guideline, and relevant recommendations were implemented in the study analysis as much as possible in the sense of methodological strictness and quality. The current review analyzes studies containing quantitative, qualitative, and mixed data and results, i.e., it represents a systematic mixed study overview (19).

### 2.1. Search strategy

Literature search for this systematic review took place between August and October 2023 and initially included the use of 3 electronic databases: PubMed, PsychINFO, SPORTdiscus. The rationale for using these databases relates to their prominent usage in other review articles (1, 14). Database searches included the following search strings (e.g., for PsychINFO): ([TITLE-ABS-KEY (sport*)] AND [TITLE-ABS-KEY (athlete*)] AND [TITLE-ABS-KEY (mental AND health)] AND (TITLE-ABS-KEY (coping strategies OR skills) AND (TITLE-ABS-KEY psych* AND problem OR stress).

The following process was applied to identify documents relevant to this systematic review: (1) Search in 3 electronic databases (PubMed, PsychINFO, SPORTdiscus); (2) hand search; (3) complementary search in Google Scholar to ensure that no studies were overlooked.

Table 1 shows the development of search terms.

**Table 1:** Development of search strings.

	Term 1	Term 2	Term 3	Term 4
General term	Mental health	Coping strategies	Elite athlete	Stress
Aspects of the general term	Well-being		Physical and mental performance	Imbalance between demands and resources
Additional aspects	Absence of illness Mental ill health	Risk factor compensation	Actively participating in competitive sports	various stressors and lack of resources leading to imbalance
Synonyms		Coping resources, Coping skills	Elite athlete, professional athlete, high-performance athlete	Pressure, tension

To minimize the risk of missing relevant studies the publishing date was not restricted.

### 2.2. Inclusion and exclusion criteria

The author analysed all matches based on title or abstract. If the information contained therein did not suffice, the relevant full text was consulted, as well. All studies included had to meet the following criteria:

• Elite athletes were defined as either members of the Olympic squad, or as participants in competitions at the international and national levels, or as being active at a professional level (e. g. NCAA) (20),
• The studies examined the effect of coping strategies on mental health,
• The contributions were original articles,
• The studies were available in full text,
• And they were published in English.

Studies were excluded from this review if

• The test persons were under the age of 12,
• The test persons did not comply with the definition of an “elite athlete” as specified in the inclusion criteria,
• Only the abstract was available, but not the full text,
• Data was missing on the study population or main findings and outcomes
• And the studies were cross-sectional because they did not allow any deductions pertaining to the effect of coping strategies preferably on mental health (lack of causality)/if the relationship between coping strategies to manage stress and outcome was not analyzed.

**Figure 1:**
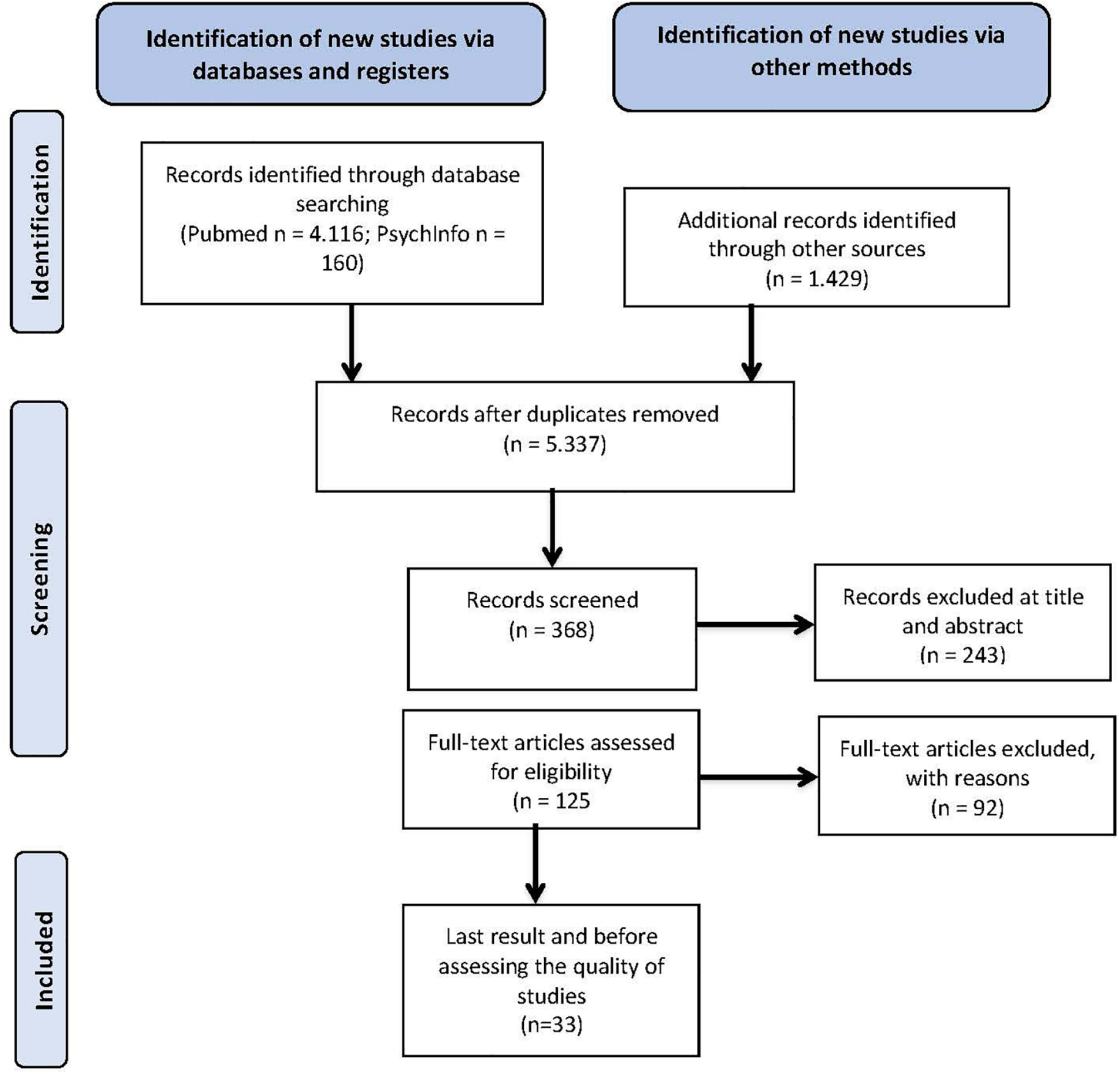
Study selection flow chart [author's own representation based on Page et al. (21)].

This systematic review followed the PRISMA guidelines (Preferred Reporting Items for Systematic Reviews and Meta-Analyses) (see Figure 1 study selection flow chart).

A template was created for data extraction to specify the required information (study design, participants, test objective, gender distribution, tests/material used, main results, key effects) of the studies included.

### 2.3. Quality assessment

Due to the heterogeneity of the studies included and due to the lack of randomized, controlled trials, a standardized risk-of-bias evaluation was not performed. As an alternative, the quality of the studies selected was evaluated based on the assessment tool “QualSyst” (22). This assessment tool includes 14 items (see Supplementary Table S1). The scoring is based on how well a scoring was met (no = 0, partial = 1, yes = 2); “NA” represents items that do not apply to the study design and are thus excluded from the calculation of the summary score. Each study's summary score was calculated by adding the total score and dividing it by the total possible score. The scores ≤55%, 55%–75%, and ≥75% indicate low, medium, and high quality, respectively. It is recommended to exclude any low-quality study from the systematic review.

## 3. Results

### 3.1. Selection of literature

There were initially 5,705 hits from 3 electronic databases (PubMed, PsychINFO, SPORTdiscus), hand search and from a complementary search in Google Scholar. All duplicates were removed, and after screening the title and abstract, the entire text was read. 33 research articles were chosen for this systematic review (see Figure 1). However, 3 out of 33 research articles were deemed to be of low quality and thus were excluded from this review. Thus, this systematic review was based on 30 studies that covered the research questions. Table 2 represents the details of the 30 studies included in this systematic review. The author has used quantitative and qualitative study designs as well as a combination of both to examine coping strategies and the effectiveness of the coping strategies applied to handle stress and to influence mental health.

**Table 2:** Overview of publication details.

Authors	Study design	Purpose	*N* (Female/Male)	Sport; Country	Coping strategies	Main findings/effects on measures of mental health
Leprince et al. (23)	Semi-structured Interview, qualitative design	Exploring stressors and communal coping strategies within **team sports**	10 team sport athletes (7 males, 3 females, age 26.3, ±7.67)	Team sports athletes (volleyball, football, basketball, ice hockey, rugby, France	Problem-focused strategies	Insight into the nature of communal coping in team sport and performance setting
Guo et al. (24)	Laboratory test, multiplication estimation task; single-factor inter-dependent design	Exploring the neural activity of the cerebral cortex under acute psychological stress in athletes with different CSE levels	106 high-level basketball players	Basketball, China	Behavioral- and task-oriented strategies	High CSE athletes can better cope with stressful events, adjust their behaviors in a timely manner according to the results of their coping and focus more on processing positive information
Hill et al. (25)	Quantitative design	Examination whether different coping tendencies mediate the relationship between self-orientated and socially prescribed perfectionism and burnout	206 junior elite athletes, age, *M* = 15.15, SD = 1.88	Judo, swimming, track athletics, field athletics*,* United Kingdom	Avoidance-focused strategies	Higher levels of socially prescribed perfectionism were related to higher levels of avoidant coping which was in turn related to higher levels of athlete burnout; higher levels of self-orientated perfectionism were related to problem-focused coping and lower levels of avoidant coping which was in turn related to lower levels of athlete burnout
Gan et al. (26)	Factor analysis	Examining the extent to which sources of stressful events and cognitive appraisal of those events predict coping style and determine differences in coping style between Chinese college elite athletes and non-elite athletes	138 elite athletes (males, *n* = 64 females, *n* = 74), 253 non-elite athletes (males, *n* = 193, females, *n* = 60) ranged from 18 to 66 years, *M* = 25,99, SD = 9,95	Volleyball, basketball, table tennis, track-field, Chinese martial arts and weightlifting, China	Approach- & avoidance focused strategies	Three sources of stress and two cognitive appraisals were significant predictors for athletes’ coping styles. Coping self-efficacy has a positive mental health effect on athletes’ ability to cope with stress.
Thelwell et al. (27)	Semi-structured interview	Examination of sources of stress and associated coping strategies	9 male professional cricket batters	Cricket, England	Problem- & relationship focused strategies	A total of 25 general dimensions for the sources of stress and 23 general dimensions for the coping strategies
Crocker and Graham (28)	Correlation analysis	Evaluation of patterns of coping, relationships between coping and negative and positive affect, and gender differences in coping and affect	235 elite athletes (males, *n* = 123, females, *n* = 112), *M* = 20.4 years, SD = 2.5	Football, volleyball, hockey, basketball, soccer, track and field, wrestling, Canada	Task-oriented & emotion-focused strategies	Group means for coping indicated that athletes primarily used strategies, such as increasing effort, planning, suppressing competing activities, active coping and self-blame; females used higher levels of seeking social support for emotional reasons and increasing effort to manage goal frustration, males experienced higher levels of positive affect
Reeves et al. (29)	Semi-structured interviews	Examination of stressors and coping strategies among early and middle adolescents	40 male academy soccer players *M* = 14.22, SD = 2.58	Soccer, England	Problem- and emotion-focused strategies	Middle adolescents reported more stressors than early adolescents, both groups experienced both common and different stressors; middle adolescents reported a greater number and repertoire of coping strategies than early adolescents, and used more problem- and emotion-focused strategies but fewer avoidance strategies than early adolescents
Bernacka et al. (30)	Prospective study	Investigation whether the personality dimension of conformism/nonconformism was a predictor of stress coping styles in athletes training combat sports, and to present the characteristics of this personality dimension in the context of competitors’ adaptive/innovative sport performance	346 males, *M* = 22, SD = 3.5	Combat sports (kick boxing, MMA, Thai boxing, boxing, and wrestling), Poland	Task-oriented strategies	Differences in stress coping styles between conformists and nonconformists training combat sporst were found as nonconformists tended to prefer task-oriented coping style
McLoughlin et al. (31)	Mixed-method design	Examination whether cumulative lifetime stress predicted depression, anxiety, and well-being in elite athletes, and to explain why cumulative lifetime stress exposure might have resulted in poor mental health and well-being	95 elite athletes (*M* = 29.81, SD = 10.88	Various sports (swimming, athletics, triathlon, football, netball, hockey, powerlifting, ultrarunning)	Various strategies	Hierarchical regression analysis revealed that total count and severity of lifetime stressor exposure significantly predicted greater depression and anxiety symptoms, and worse well-being; thematic analysis revealed that cumulative lifetime stress exposure fostered **poor mental health** and well-being by promoting maladaptive coping strategies
Anshel et al. (32)	Multivariate analysis of variance	Examination of sources of stress, which are performance or coach related and respective coping styles, depicted as approach and avoidance, as functions of both gender and race	332 (males, *n* = 176 females, *n* = 156), *M* = 21.6, SD = 4.86	Various sport disciplines, African Americans, Caucasians, and Hispanics	Approach behavioral and avoidance oriented strategies	Caucasians experienced higher stress intensity and tended to use only an approach-behavior style; women reported higher stress intensity for coach-related sources of acute stress and used approach-behavioral and avoidance coping styles more often than their male counterparts; Hispanics did not differ from other groups on any measure
Skein et al. (33)	Pre-post design	To examine sleep characteristics, scheduling of activities, perceived stress and coping strategies between periods of high and low scheduling commitments in adolescent athletes	20 Australian adolescent athletes (males, *n* = 10 females, *n* = 10), *M* = 15.0, SD = 1.0	Netball, rugby league, basketball, softball, and athletics, Australia	Behavioral and task oriiented strategies	Stress levels were significantly incresed in periods of high scheduling commitments with no differences between sexes Chronic sleep deprivation experienced by elite athletes may suggest that not only their recovery is impaired but also their mental health and well-being may be negatively affected.
Kristiansen and Roberts (34)	Qualitative design, question focused analysis	To examine how young athletes experienced competitive and organizational stress and how they coped with these stressors	29 young Olympic athletes, males, *n* = 8, females, *n* = 21, age, *M* = 16.6, SD = 0.77	Track and field, basketball, cycling, gymnastics, handball, judo, swimming, table tennis, volleyball, water polo, Norway	Cognitive strategies	Athletes experienced competitve stressors because of the size and importnace of the competition, and organizational stressors because of the extrme heat during the competiton, athletes used cognitive coping strategies in addition to relying on different types of social support Also, unsuitable coping strategies, insufficient support, and lack of competition experience were identified as stressors and are therefore to be considered risks, especially to mental health
Litwic-Kaminska (35)	pre-post design	To distinguish different types of sport competition appraisals and to verify if athletes’ interpretation of a stressful situation changed their choice of coping methods	193 athletes (males *n* = 97, females *n* = 95), age *M* = 20.27, SD = 3.51, following Summer Olympic disciplines	Shooting, handball, rowing, judo, taekwondo, volleyball, football, Poland	Cognitive strategies	Three types of of sport competition analysis were identified: positive, negative and active; participants who revealed positive appraisals undertook the highest number of actions aimed at reaching goals and least frequently sought support
Pensgaard and Ursin (36)	Mixed-method design, observational	To explore the different dimensions of the stress experience and the following coping efforts among elite athletes	70 Winter Olympic athletes, males, *n* = 50, females, *n* = 20, *M* = 25.2, SD = 3.8	Various winter sports, Norway	Problem-focused and cognitive-defense strategies	Stress was mainly experienced during the time period prior to the competiton; external distractions and expectations were the most frequently reported stress experiences; the coach was seen as a major source of stress by some athletes; problem-focused coping strategies were employed at all times while cognitive strategies were used more days before and after the competition
Yi et al. (37)	Correlation analysis	To examine whether resilient group would differ from nonresident group in using more adaptive coping strategies (e.g., problem-focused coping, seeking social support, rational reappraisal to minimize threat) and would be correspondingly less likely to favor the potentially maladaptive ones (e.g., blaming others, avoidance, wishful thinking; to further examine any interrelations between these two groups regarding the use of coping strategies	404 young women high school athletes (*M* = 15.76, SD = 1.08	Basketball, gymnastics, cross-country, soccer	Problem-focused strategies, seeking social support and avoidance strategies	Coping profiles of the two groups differed significantly, with resilient athletes favoring problem-focused coping and seeking social support, and nonresilient athtletes reporting greater use of avoidance and blaming others; correltations among problem-focused coping, seeking social support, and minimize theat were higher in the resilient groupt
Szczypińska et al. (38)	Online-survey including four psychological questionnaires	To compare strategies of coping with stress during the COVID-19 epidemic in athletes involved in Olympic preparations and students of physical education	First group: 57 potential Olympians practicing individual sports, *M* = 26.6, SD = 5.562; second group:54 extramural students of physical education, *M* = 25.69, SD = 5.908	Athletics, rowing, fencing, shooting, sport climbing, badminton, swimming, modern pentathlon, taekwondo, sailing, wrestling, canoeing, judo, cycling, equestrianism and weightlifting, Poland	Cognitive and behavioral coping strategies	Elite athletes and physical education students practicing sports most often dealt with the stress of the COVID-19 pandemic using cognitive and behavioral coping strategies; the sports level depended on the strategies of coping with stress more strongly than gender
Secades et al. (39)	pre-post design	To analyze the relationship among resilient qualities and coping strategies	235 athletes, males, *n* = 126, females, *n* = 109, age, *M* = 20.7, 79.1% team sports, 20.9% individual sports	Various team sports (soccer, handball, volleyball, rugby) and individual sports (gymnastics, triathlon, athletics, cycling), Spain	Emotion-oriented strategies, task-oriented and, disengagement strategies	No significant difference in resilience scores between evaluations performed during the last mesocycle or competition; a significant increase occurred in the scores for emotion-oriented and distraction-oriented coping during competition; resilience scores correlated positively to task-oriented and negatively to disengagement and distraction-oriented coping during both periods; athletes with high individual resilient qualities reached higher scores in task-oriented coping, using to a lower extent disengagement- and distraction-oriented coping
Fogaca (40)	Pre-post design	To teach college student-athletes coping skills to improve both performance and mental health and increase their social support from coaches and captains	88 college-student athletes (51% female, 83% white), *M* = 19.8, SD = 1.1, intervention and wait-list control groups	Soccer, basketball, golf, swimming, and diving, United States	Task-orientied strategies	Athletic coping skills and anxiety significantly improved for the intervention group, compared to the control group The present findings indicated that ineffective coping increased the athletes’ negative affect and cognitions, triggering physiological and psychological responses
Nicholls et al. (41)	Prospective design	To examine a model, informed by self-regulation theories, which included goal adjustment capacities, appraisals of challenge and threat, coping, and well-being	212 athletes (team sports, *n* = 135, individual sports, *n* = 77, males, *n* = 107, females, *n* = 105) age, *M* = 18.96, SD = 5.74	Team sports (soccer, rugby union or rugby league), individual sports (tennis, golf, martial arts), United Kingdom and Australia	Task-oriented strategies	The way an athlete responds to an unattainable goal is assoiciated with her or his well-being in the period leading up to and including the competition; goal reengagement positively predicted well-being, whereas goal disengagemnt negatively predicted well-being; goal reengagement was positively associated with challenge appraisals, which in turn was linked to task-oriented coping, and task-oriented coping positively associated with well-being
Cumming et al. (42)	Hierarchical regression analyses	To examine relations among body size, stress coping strategies, and mental health	44 female high school students, age, *M* = 15.8, SD = 1.1	Basketball and gymnastics, Caucasian (*n* = 134), African Americans (*n* = 6) and Asians (*n* = 9)	Adaptive vs. maladaptive strategies	The use of adaptive over maladaptive coping strategies was associated with more positive mental health and less distress in both sports; in gymnasts, BMI was inversely related to psychological well-being and the interaction between height and less adaptive coping strategies significantly predicted psychological distress
Krokosz and Jochimek (43)	Correlation and multiple regression analysis	To analyze relationships between strategies of coping with stress used by male and female extreme athletes, perception of threat associated with their sport, and their satisfaction with life	144 athletes, males, *n* = 89, females, *n* = 55, age, *M* = 23.85, SD = 5.78	Poland, watersports (kitesurfing, windsurfing, wakeboarding, freediving, BMX, roller skating, extreme scooter, skateboarding, motocross, downhill mountain biking, parkour)	Emotion-oriented & behavioral strategies	Significant relastionships were found between the use of certain strategies for coping with stress and the assessment of risks associated with extreme sports in both men and women; only in the case of men were relationships observed between coping strategies used and satisfaction with life; women were more likely to use emotional and instrumental support and less likely to use humor than men
Deroche et al. (44)	Hierarchical regression analysis	To examine whether pain coping strategies, including distraction from pain, praying, reinterpreting pain sensations, ignoring pain, pain catastrophizing, are related to athletes’ inclination to play through pain	205 athletes, males, *n* = 158, females, *n* = 47, age, *M* = 22.73, SD = 6.45	Combat sports (judo, taekwondo, karate, and wrestling)	Behavioral strategies	Pain catastrophizing led athletes to reduce their physical involvement in their sport activity; moderating effect of ignoring pain such that ignoring pain significantly attenuated the negative effect of pain intensity on on athletes’ inclination to play through pain
Kerdijk et al. (45)	Diary study, qualitative design	To examine whether the social environment (significant others) is of influence on the stress and coping of team athletes	6 athletes, males, *n* = 2, females, *n* = 4, *M* = 23.0, 6 team athletes of different sports, males, *n* = 2, females, *n* = 4,, *M* = 25.8	Belgium, Netherlands, hockey, soccer, futsal, cricket	Problem-, emotion- and avoidance focused strategies	In particular teammates are important for the appraisal of stress and coping in teamsports; team athletes experienced the highest stress intensity during competition, or when they appraised the situation as a threat; when others were of influence the team athletes were most likely to appraise the situation as a challenge and use problem- or emotion-focused coping strategies
Dolenc (46)	Multiple regression analysis	To examine self-esteem, anxiety level and coping strategies among secondary school students in relation to their involvement in sports	280 secondary school students, males, *n* = 140, females, *n* = 140, two groups, athletes, *n* = 140, *M* = 16.6, SD = 1.1, non-athletes, *M* = 16.7, SD = 1.2	Slovenia, various sports	Problem-focused strategies	Athletes exhibited higher self-esteem scores and lower anxiety scores in comparison to non-sport participants; differences between the two groups have also been identified with respect to the use of certain coping strategies; sport participants reported an active and problem-focused approach to dealing with everyday problems; gender diffrences have also been studied with female athletes exhibting higher levels of anxiety than male athletes; female participants were also found to use more non-productive coping than males, focused mainly on reducing emotional effects of stress
Daumiller et al. (47)	Online survey Regression analysis	To examine the effects of elite athletes’ achievement goals on their burnout levels and psychosomatic stress symptoms, and to what extent they can be explained by athletes’ use of adaptive coping strategies	165 elite athletes, males, *n* = 97, females, *n* = 28, *M* = 23.7, SD = 4.0	Football, badminton, handball, gymnastics, ice hockey, skiing, basketball, volleyball, swimming, boxing, squash, tennis Germany	Approach and avoidance oriented strategies	Path modelling revealed that mastery approach goals were negatively associated with burnout levels and psychosomatic stress symptoms, while mastery avoidance and performance approach goals were positively associated with burnout levels; coping stregies partially mediated the effects of mastery approach goals on burnout levels and psychosomatic stress symptoms
Anshel and Anderson (48)	Test battery	To test the extent to which highly skilled table tennis players used coping strategies that were consistent with their coping style, both of which were categorized as approach and avoidance, in response to performance-related sources of acute stress on a table tennis task	36 male elite athletes, *M* = 32.0, SD = 1.67	Australia, table tennis	Approach and avoidance oriented strategies	Primary results indicated significant correlations between the athletes’ approach and avoidance coping styles and their respective use of coping strategies; an approach coping style was a significant predictor of performance on the first block of 20 trials, whereaas a combination of positive affect, avoidance coping strategies and negative affect best predicted performance on the second block of 30 trials
Raedeke and Smith (6)	Structural equation modeling, hierarchical multiple regression analysis	To examine whether coping behaviors and social support satisfaction had indirect stress-mediated or moderated influences on athlete burnout	244 senior age-level athletes, males, *n* = 112, females = 131, *M* = 15.8, SD = 1.3	United States, swimming	Behavioral and relationship-oriented strategies	Results demonstrated that general coping behaviors and social support satisfaction had stress-mediated relationships with overall burnout levels; hierarchical multiple regression analysis failed to support the disjunctive and conjunctive moderation hypotheses
Britton et al. (49)	Path analysis	To examine a path analysis of adolescent athletes’ individual differences in perceived stress reactivity, competition appraisals, emotions, coping and performance satisfaction	Adolescent athletes (*n* = 229, males, *n* = 150, females, *n* = 79), *M* = 18.55, SD = 2.40	Team sports (rugby, football and cricket) and individual sports (golf, karate and badminton), United Kingdom	task-orientated strategies	A path analysis revealed that perceived stress reactivity had direct and indirect effets on the appraisal of higher stressor intensity, lower perceived control, higher perceived threat, positive and negative emotions and maladaptive coping were associated with performance satisfaction; task-orientated coping was not associated with performance satisfaction
Nicholls et al. (50)	Data was collected using concept maps (51), which were used as an open-ended questionnaire	To examine stressors, coping and coping effectiveness as a function of gender, type of sport and skill	749 undergraduate athletes (*n* = 455, males, *n* = 294, females), 217 = individual sports 532 = team sports *M* = 19.8, SD = 2.33	Individual sports (e.g., badminton, golf, martial arts and swimming), team sports (e.g., basketball, cricket, football, hockey and rugby), 711 Caucasian, 20 Black and 18 Asian athletes	Problem-focused strategies, task-oriented strategies	The results revealed gender, type of sport and skill differences in relation to stressor frequencies, coping strategy development, and coping effectiveness; females used more freqeuntly problem-focused (e.g. planning, communication, technique-orientated) strategies; team sport athletes reported a variety of sport-specific stressors relating to the demand of playing in a team environment; the group of national/international athletes reported using more planning, blocking and visualization and reported that their coping was more effective than that of less-skilled athletes
Goyen and Anshel (52)	Quantitative design	To examine sources of acute stress and related coping processes following stressful events in competitive sport	Adolescent-aged athletes, *n* = 74 (*n* = 39, males, *n* = 35, females), *M* = 15.4, SD = 1.61; adult-aged athletes, *n* = 65 (*n* = 37, males, *n* = 28, females), *M* = 26.6, SD = 2.26;	Various team sport disciplines (netball, soccer, cricket, hockey, rugby union, basketball), Australia	Problem- & emotion-focused strategies	Chi-square analysis indicated significant age and gender differences in the frequency with which selected coping strategies were used as a function of the stressor; males preferred problem-focused coping, females used emotion-focused coping after the stressors; younger athletes use more maladaptive coping skill more often than older-aged competitors, they are more susceptible to both acute and chronic forms of stress in sport

Interestingly, the first study included was published in 1998 (Pensgaard & Ursin), which emphasizes a constant interest in coping in an elite sporting context. In addition, most studies have focused more on coping to handle stress rather than comprising the impact of coping on elite athletes' mental health (31, 40, 47).

The analyzed study results point to a broad spectrum of coping categories, elite athletes make use of to deal with stressful situations. The results representation outlines a categorization of the most widely used higher-order coping dimensions based on their function and intension (12, 53), such as problem-orientated and emotion-regulative coping. Problem-orientated coping strategies are intended to change the stressful situation, whereas emotion-regulative coping deals with the emotional distress associated with the situation (10). To describe the complexity and heterogeneity of coping within the context of elite sport and the effectiveness of coping strategies applied by elite athletes regarding their mental health, some higher-order coping dimensions were used to structure the study results. Avoidance coping comprises both behavioral and psychological efforts to get rid of a stressful situation (54). Approach coping strategies involve the confrontation of the source of stress and the attempt to reduce it (e. g. taking direct action or planning) (55). With the help of appraisal-focused coping the stressful situation is re-evaluated to reduce its intensity and importance (e. g. re-structuring) (56). The application of the aforementioned macro-level dimensions of coping seems to be suitable to provide an overall characterization of elite athletes' stress responses. However, these higher-order dimensions conceal the heterogeneity and complexity of the various coping responses and consider insufficiently the aspect of mental health. As the extracted literature mainly focused on coping in elite sporting context the classification of coping was completed by (1) type of sports, (2) gender, (3) dimensions of coping and (4) effectiveness of coping in terms of mental health. This approach was chosen to refer best to the review's objective/research questions.

### 3.2. Coping and type of sports

Findings show that teammates play an important role in the experience and appraisal of stress and coping in team sports.

When others influenced the stress and coping process situations were more likely to be appraised as a challenge and more adaptive problem- and emotion-focused strategie**s** were used, whereas when a stressor was appraised as a threat a more maladaptive coping strategy or no coping at all was reported (23, 45). The results also revealed that there is a significant relationship between coping with stress and perceptions of risk in extreme athletes, practicing extreme sports (43).

Pain coping strategies including distraction from pain, praying, reinterpreting pain sensations, ignoring pain and pain catastrophizing were observed to be related to athletes' inclination to play through pain (44).

The results indicated that pain catastrophizing explained an incremental portion of the variance in the prediction of sport-related pain behavior, over and above the intensity of pain experienced during sport activity. The more athletes catastrophized their pain, the less they were inclined to play through the pain.

### 3.3. Coping and differences in gender

Regarding gender the analyzed studies revealed some differences. Studies observed that female athletes showed higher levels of anxiety and used more often emotion-focused coping strategies compared with male athletes and that female athletes experienced more intense stress from their coaches than did male athletes (32, 46). Also, skill level affected athletes' coping style among both female and male athletes; according to the percentages, more elite male athletes had an approach coping style, and more non-elite male athletes had an avoidance coping style, and more elite female athletes had an avoidance coping style and more non-elite females had an approach coping style. As well, findings showed that there are significant differences between men and women in their assessment of the frequency of using given strategies for coping with stress. Women were more likely than men to use strategies of searching for emotional support and instrumental support in threat situations; men reported using the strategy of relying on a sense of humor significantly more frequently than women did. No statistically significant differences between men and women were observed in levels of satisfaction with life and in the assessment of risks associated with extreme sports.

### 3.4. Coping strategies applied in elite sports

Study results regarding coping strategies in elite sports to handle stress showed that athletes adopted more often a task-oriented coping style to confront stressful sport-related situations (30, 36).

Investigation of coping strategies in relation to athletes' illness revealed significantly different profiles of coping in resilient and non-resilient athletes. Those athletes, who remained healthy despite an elevated level of recent life stress generally favored problem-focused coping and seeking social support, whereas in the non-resilient group avoidance strategies were more dominant (37). Results also indicated that resilience correlated positively with task-oriented coping and negatively with emotion-oriented coping. Participants with lower resilience scored higher on emotion-oriented coping and distraction-oriented coping, whilst higher scores on resilience were observed on task-oriented coping (39). The findings of Hill et al. (25) revealed that avoidant coping was related to higher levels of athlete-burnout and higher levels of problem-focused coping were related to higher levels of self-oriented perfectionism.

Studies concerning coping style and coping strategies showed that athletes often used coping strategies that were commensurate with their coping style.

### 3.5. Effectiveness of coping in terms of mental health and handling stress

The results of Dolenc (46) showed that problem-focused coping is associated with lower levels of anxiety and that emotion-focused coping is associated with higher levels of neuroticism. The obtained results indicated positive correlations between proactive coping, moderate physical activity, self-efficacy, and mental health. Results indicated as well that biological and psychological factors contribute in complex ways to influence the psychological well-being and mental health status of young female athletes. Regarding the use of coping styles, the balance between adaptive and maladaptive coping styles predicted an outcome variable of clinical significance (42).

Coping strategies emerged as a predictor of variance of mental health in gymnastics but not in basketball. The results of the present study revealed that the adaptive scales were positively related to well-being and negatively to distress. The observation that coping style was most closely associated with mental health in comparatively short gymnasts was novel and of particular interest.

The study of Guo et al. (24) examined differences between athletes with high and low coping self-efficacy (CSE) levels under acute psychological stress*.* The study showed that low-CSE athletes lacked confidence and hence were more likely to not know what to do when facing stress. By contrast, low-CSE athletes paid greater attention to negative information and the consequences of failure, not only causing them to lose confidence in their abilities but also affecting their subsequent coping behavior and possibly their mental health status.

The results showed that athletes needed to be prepared for the total competitive experience that includes both organizational and competitive stressors. Further findings revealed the importance of social support as a coping strategy and that informational and emotional support in combination with cognitive strategies were used most to cope effectively with competitive stress and to influence mental health positively. In addition, coach support was important to cope with both organizational and competitive stressors and it turned out that athletes tend to seek more likely support from people the feel close to.

## 4. Discussion

This systematic review attempts to outline the current knowledge of coping strategies for handling stress and providing mental health in elite athletes.

Overall, the findings of this review add to domain-related reviews (1, 14) suggesting, that elite athletes face various stressors during their sports career, that, if not well managed, put them at an increased risk of poor mental health. Despite growing interest in this topic, the factors that influence elite athletes’ mental health remain unclear and therefore warrant further research. The transactional model of stress and coping (10) proposes that effective coping is an important variable to buffer the detrimental effects of stressors on mental health, as coping resources theoretically influence an athlete's ability to effectively deal with the challenges and stresses of sport participation.

According to Lazarus (57) coping strategies can be described as behaviors that help elite athletes to handle their problems, joys, and stresses of life. In line with Hobfoll's COR theory (58) someone with resources is less likely to encounter stressful circumstances that negatively affect psychological well-being and mental health. Hence, an elite athlete with coping skills such as high coping self-efficacy, highly pronounced resilience, sense of coherence or support from coaches, teammates, family members or friends can apply these resources in terms of coping with stressful situations towards growth and development instead of using these skills defensively to offset stressors. The athlete makes use of his resources to solve problems inherent to stressful situations. The more resources are available the more likely these resources are used to fit upcoming demands in stressful contexts. However, limited attention has been given to the potential relationship of athletic coping skills with well-being and mental health and how coping and its effectiveness are associated with elite athletes' mental health, despite Smith et al.'s (59) suggesting that these variables would be related to each other.

According to the current state of research stress symptoms, stemming from physical as well as psychological factors, are especially detrimental to elite athletes, as they rely on their physical and mental health and functioning for performance success. An increasing body of evidence shows that elite athletes experience mental-health problems, that can be maladaptive if not treated at an early stage, as they can lead to compromised psychological and physiological functioning (60). Concerning the relationship between stress and resulting mental health problems, the different coping strategies elite athletes use to handle stress can be important (15, 61).

Therefore, dealing effectively with stress and the actions one decides to take depend mainly on the characteristics of the stressor and largely determines the health costs of a stress transaction (62).

This systematic review aimed to address this issue by analyzing both quantitative, qualitative, and mixed-method study designs examining elite athletes and the effectiveness of their coping strategies to handle stress and to protect their mental health. The findings are also consistent with the transactional model (10, 57) and show parallels to the biopsychosocial determinants (see Table 1) influencing mental health in an elite sporting context (63). Worth mentioning are determinants on the psychological level (e.g., personality, perceived stress, coping repertoires) as well as on the social level (e.g., support systems) and the biological level (e.g., sleep, gender, age) as they were all focused on in the studies analyzed in this review.

### 4.1. Coping and type of sports

The identification of new coping strategies at the team sports level represents an important step in the understanding of communal coping in team sports because it offers a new perspective of how teams cope dealing with communal stressors. The identified communal coping strategies concretely describe the collective actions teammates use to cope.

The findings of studies analyzed in terms of type of sport showed that in extreme sports concentrating on forming interpersonal relations through looking for instrumental and emotional support seems more natural to women (43). One possible explanation could be that women assess threats more realistically and do not feel the need to underestimate them to gain social approval. Men in contrast tried to preserve their image as brave and strong individuals.

Competing despite having health problems can cause irreversible physical damage and therefore may affect not only an athlete's professional career but also his long-term health. Studies investigating the phenomenon of playing hurt have shown that playing despite having health problems often is accompanied with disregarding medical guidelines or hiding pain from coaches and teammates (7). As social pressure is mainly generated by coaches or other team members, it can be presumed that also the way of communication in sports networks plays a decisive role regarding athletes’ willingness to compete hurt or ill. In this regard the role of the coach regarding establishing athlete-centered communication strategies to prevent athletes from pain-trivializing or competing despite injuries or illness seems to be more than mandatory (64).

The present review emphasized the contribution of pain coping in predicting athletes' inclination to play through their pain, whereas previous studies have shown how social networks can lead athletes to accept pain as a “part of the game” and can generate pressure on athletes to continue competing despite such pain (65, 66).

### 4.2. Coping and differences in gender

The findings of the analyzed studies show that there may be various reasons for gender differences in coping behavior (32, 46).

Findings are also consistent with previous sport psychology research showing that female athletes assess stressful situations as more negative compared to male athletes. Female athletes had stronger feelings of tension and worry with greater susceptibility to a variety of stressful events and seek for more social support. One reason that males and females cope differently could be the emergence of differences in the socialization process because of gender role stereotypes and expectations from the social environment. That could implicate that gender differences may influence an athlete's selection of coping styles and strategies and, that females are more inclined to make use of an emotion-oriented coping style, whereas men use more often active coping. Also, the significant findings between skill levels for the athletes' use of cognitive appraisal and coping style are consistent with results of former studies (67). There is to some extent empirical evidence that less-skilled male and female athletes differ on various psychological characteristics. However, differences in psychological characteristics based on gender are less common among highly skilled athletes (68). It is possible, that at a particular elite level of competition, athletes of both genders make cognitive appraisals and adopt coping styles that are similar and effective.

### 4.3. Coping dimensions applied in elite sports

In view of the coping dimensions applied in elite sports the findings of this review suggest that among the analyzed strategies of coping with stress, avoidance may be related to health and absenteeism by contributing to psychological distress and emotional arousal, which may in turn suppress immunity. In addition, there is evidence that avoidance could also relate to illness in terms of not taking care of oneself when one is ill (38). As illness itself could serve as a socially accepted means of avoiding stressful situations, an avoidance strategy may temporarily be adaptive to cope with uncontrollable stressors. One possible disadvantage could be that illness prevents the use of more problem- and emotion-focused strategies in terms of proactive coping. Furthermore, the willingness to use active coping strategies can be predicted on the base of meaningfulness and sports activity. It can be expected that problem-focused strategies are more likely used by athletes or people with a strong sense of meaningfulness.

According to Aspinwall and Taylor (69) proactive coping involves providing the necessary resources and skills to prepare for confronting and anticipating stressors.

As resilience is one of the resources permitting athletes to protect their health (16), resilience takes on great importance to determine whether athletes will be able to go through stressful situations they are confronted with during their sports career to cope with their stressors. According to the findings it seems that high resilient qualities associate with coping strategies that might contribute more effectively to adapt to stressors and possible failures in elite sports (39). However, more research is required to confirm that enhanced resilience might not only contribute to better sport performance but also improve elite athletes' coping skills and mental health status, as resilience is a relatively stable construct (70).

### 4.4. Effectiveness of coping in terms of mental health and handling stress

The accumulation of stressors during childhood was found to render participants more susceptible to stress throughout adulthood. This finding suggests that the accumulation of stressors over life course may limit the coping resources to deal with the demands of a stressful situation. When coping resources are limited, individuals will typically appraise a stressful situation more as a threat. Repeated threat appraisals have been linked to deleterious health consequences (e.g., depression). Athletes who experienced greater and more lifetime stressors demonstrated difficulty in establishing and maintaining interpersonal relationships. According to extent literature a lack of interpersonal relationship can increase the cumulative effects of stress (31).

The analysis of the reviewed papers goes in line with Lazarus' (61, 71) findings that emotions will occur when a person appraises encounters with the environment as having either a positive or negative impact for well-being in terms of the person's goals. Fear will be provoked when an individual believes that failure is a threat to the achievement of goals.

Chronic sleep deprivation experienced by elite athletes may suggest that not only their recovery is impaired but also their mental health and well-being may be negatively affected.

For athletes, poor sleep can result in impaired recovery, but also diminished athletic performance and increased injury risk are seen as health influencing factors. According to Asplund and Chang (60), a bi-directional relationship has been observed, which means that sleep problems in athletes are positively correlated with depression or anxiety, while just mentioned psychiatric conditions can lead to sleep disturbances and therefore compromise sleep quality. Coming along that due to fear of negative evaluation, lack of mental health literacy and/or limited knowledge of where and how to seek help, elite athletes still fail to report sleep and mental health problems (72).

Aside from these barriers mentioned above and differential sport demands there may undetermined athlete differences that influence the nature of coping responses. Differences in psychological maturity may explain why the tendency to employ adaptive vs. maladaptive coping strategies was more closely associated with mental health in athletes. Especially, if athletes are delayed in their psychological maturity, they may be less capable of using adaptive coping strategies to effectively deal stressors, including sport-specific demands, such as participating at competitions, training, and social life.

Again, the findings are reflected in the research of Lazarus and Folkman (10). From their point of view individual resources that help to deal successfully with the demands imposed by of a particular situational context (in our case the context of elite sports) determine the level and intensity of distress experienced. That's why resources should be directed to detect early signs of mental health problems in this age group, as around 19 years of age, just after completing high school and moving from junior to senior competitions, seems to be a particularly vulnerable age for onset of mental health problems in elite athletes.

The results of this review underpin the necessity that especially college-students being confronted with a wide range of stressors, need to be taught mental skills to cope with these stressors. In addition, teaching coaches and teammates about social support seemed to decrease college-student athletes' stress reactions, such as anxiety or depressive symptoms. This finding is also in line with the transactional model of stress and coping (10), which proposes that coping efforts and social support can decrease the effects of stressors on a person's mental health.

However, traditional sport psychology is mainly focused on optimizing performance in a presumably healthy population, although mental health problems in elite sport have received increased attention, revealing the need for a broader mental health continuum (42, 73) and for more attention to the potential relationship of athletic coping skills with well-being and mental health (40). As coping self-efficacy has a positive mental health effect on athletes' ability to cope with stress, athletes can thus better cope with and eliminate interference caused by competitive stressors and eventually achieve their best performance and protect their mental health (24).

### 4.5. Practical implications and future directions

Understanding the mechanisms of the coping process and its complexity in the context of handling stress may assist in the development for future interventions. As mental health plays a key role in many functions that are necessary in a context of elite sporting, future studies may investigate strategies to cope effectively with the stressors elite athletes are confronted with, such as improving stress management techniques to avoid stress. To improve mental health and coping skills exploring the coping process across a broader range of sports and participants may help to reduce athletes' vulnerability to emotional abuse and any mental health impairments.

Therefore, more research is needed to determine the underlying factors and antecedents that predict an athlete's coping preferences following specific types of stressful events and then to target meaningful and effective interventions (26). With the help of longitudinal designs links between an athlete's coping style and their actual application of coping strategies following various sources of acute stress as a function of sport type (e.g., individual and team), skill level, gender, age, and culture can be examined (48). That means that future approaches in sport psychology research may move beyond the identification of stressors and prevalence rates to point more vehemently to the complexity of mental-health problems in elite sports. The research directions displayed in Table 3 could be adapted to an elite sporting context in future projects to investigate and improve understanding elite athletes' coping strategies regarding to cope with stress and mental health.

**Table 3:** Practical implications & future directions.

Practical implications	Future directions
• Athletes competing in **individual sports** or sports where athletes are expected to reach their performance peak at an early age, such as gymnastics, may benefit from learning to use adaptive coping strategies [like problem-focused coping, seeking social support, or cognitive restructuring (minimizing threat)] (42) • In **team sports** coping effectiveness training programs combined with attention modification issues should be incorporated in the training of athletes to help them to better cope with and eliminate interference caused by competitive stressors and eventually achieve their best performance and protect their mental health (24)	• Development of stress management programs to help **teams** to collectively solve problems, to strengthen the mental health status of the whole team and to strengthen relationships under stressful conditions (23) • Exploring the coping process across a **broader range of sports and participants** to reduce athletes’ vulnerability to emotional abuse and any mental health impairments,
• Relationships between **protective or immune resource**s such as the sense of coherence and the **effectiveness of coping strategies** to deal with stress in an extreme situation such as the COVID-19 pandemic should be examined in a more detailed way in the context of developing coping interventions in the context of elite sports (38)	• More research is needed to determine the underlying factors and antecedents that predict an athlete's coping preferences following specific types of stressful events and then to target meaningful and effective interventions to improve **mental health** and **coping skills** (26)
• Training adolescent athletes to use **task-orientated coping** might help them to deal with competitive anxiety in sport. • A **supportive coach-athlete relationship** is highly recommended especially if coaches want athletes continuing **long-term competitive participation** (34) • Assessing simultaneously the **use** and **effectiveness of coping strategies** to clearly disentangle the effects of the use vs. effectiveness of a wide variety of coping strategies	• Examining links between an athlete's coping style and their actual application of coping strategies following various sources of acute stress as a function of sport type (e.g., individual and team), skill level, gender, age, and culture with the help of l**ongitudinal designs** (48)

### 4.6. Limitations

This systematic review shows a few noteworthy limitations. First, this systematic review includes only English language studies, which may limit the representation of the results. Second, this review is not a meta-analysis which was not possible to realize due to the heterogeneity of study designs. Third, the paucity of research in the field and the lack of well-designed, intervention-based research in elite athletes' coping to handle stress and to provide mental health contributes the poor overall quality of study reporting on this topic (39, 40). Moreover, variables such as age, gender, type of sport (e.g., individual or team sport) or effectiveness of applied coping strategies have not been considered in a standardized manner (34, 48).

Finally, the author's attempt to structure the dimensions of coping must be considered in a critical way. According to the cognitive-transactional stress theory, two functions can be distinguished: problem-related coping and emotional coping (57). However, the function is seen independently of the effect. According to Weber and Laux (74) this dual function model should not be classified by functions, but rather by subjective intentions, i.e., by the very personal functionality of a behavior with respect to an individual's preferred goals. These personal intentions can be divided into four facets: the regulation of emotions, the solution of the underlying problem, the maintenance of self-worth, and the control of social interactions. Whether and when these four regulatory goals are met is then a question to be addressed independently. Content areas such as well-being, physical and mental health, or social behavior serve as one criterion for efficiency.

## 5. Conclusion

Coping in elite sporting settings is very complex and dynamic. This is the first systematic review that has analyzed coping strategies to handle stress and to provide mental health in an elite sporting context. The findings of this review lend increased support that an athlete's use of coping strategies at least partially reflects the coping style. The application of coping style and coping strategies seem to be a function of the type and the intensity of the stressors an athlete is confronted with or of the athlete's cognitive appraisal of the stressful event.

Results highlight that coping strategies play an important role in understanding the handling of sport-specific and non-sport specific stressors in an athlete's professional career. There is evidence of coping being effective to buffer stress, but the interrelationships between stressor, appraisal of the stressor, application of a corresponding coping strategy, its effect especially in terms of mental health outcomes is still unclear because of lacking intervention-based study designs.

Future research is needed that assesses these relationships primarily as previous studies revealed that elite athletes tend to ignore and trivialize health risks and act within a coherent culture of risk to perform successfully.

## Data Availability

The original contributions presented in the study are included in the article/**Supplementary Material**, further inquiries can be directed to the corresponding author.
