# Supplementary material for: Coping strategies for handling stress and providing mental health in elite athletes: a systematic review
Source: Front Sports Act Living. 2023 Nov 16;5:1265783. doi: 10.3389/fspor.2023.1265783 (PMC10687549; doi:10.3389/fspor.2023.1265783)
Supplement: Supplementary file 1 [file Table1.docx]

**Table 3:** “Qualsyst” quality assessment

| **Publication** | **Question/objective described** | **Appropriate study design** | **Appropriate subject selection** | **Characteristics sufficiently described** | **Random allocation** | **Researchers blinded** | **Subjects blinded** | **Outcome measures well defined and robust to bias** | **Appropriate sample size** | **Analytic methods well described** | **Estimate of variance reported** | **Controlled for confounding** | **Results reported in detail** | **Conclusion supported by results?** | **Rating** |
| --- | --- | --- | --- | --- | --- | --- | --- | --- | --- | --- | --- | --- | --- | --- | --- |
| Leprince et al., 2018 | 2 | 2 | 1 | 2 | 0 | 0 | 0. | 1 | 1 | 2 | 2 | 2 | 2 | 2 | **medium** |
| Guo et al., 2019 | 2 | 2 | 2 | 2 | 0 | 0 | 0 | 2 | 2 | 2 | 2 | 2 | 2 | 2 | **high** |
| Hill et al., 2010 | 2 | 2 | 2 | 2 | 0 | 0 | 0 | 1 | 2 | 1 | 2 | 2 | 2 | 1 | **medium** |
| Gan et al., 2009 | 2 | 2 | 2 | 2 | 0 | 0 | 0 | 1 | 2 | 2 | 2 | 2 | 2 | 1 | **medium** |
| Daumiller et al., 2021 | 2 | 2 | 2 | 2 | 0 | 0 | 0 | 1 | 2 | 2 | 2 | 2 | 2 | 2 | **high** |
| Anshel & Anderson, 2002 | 2 | 2 | 2 | 2 | 0 | 0 | 0 | 2 | 1 | 1 | 2 | 1 | 2 | 1 | **medium** |
| Britton, Kavanagh & Polman, 2019 | 2 | 2 | 2 | 2 | 0 | 0 | 0 | 2 | 2 | 2 | 2 | N.A. | 2 | 2 | **high** |
| Raedke, & Smith, 2004 | 2 | 2 | 2 | 2 | 0 | 0 | 0 | 1 | 2 | 2 | 2 | N.A. | 2 | 2 | **medium** |
| Nicholls et al., 2007 | 2 | 2 | 2 | 2 | 0 | 0 | 0 | 1 | 2 | 2 | 2 | 2 | 2 | 1 | **medium** |

**Table 3** *continued: Qualsyst” quality assessment*

| **Publication** | **Question/objective described** | **Appropriate study design** | **Appropriate subject selection** | **Characteristics sufficiently described** | **Random allocation** | **Researchers blinded** | **Subjects blinded** | **Outcome measures well defined and robust to bias** | **Appropriate sample size** | **Analytic methods well described** | **Estimate of variance reported** | **Controlled for confounding** | **Results reported in detail** | **Conclusion supported by results?** | **Rating** |
| --- | --- | --- | --- | --- | --- | --- | --- | --- | --- | --- | --- | --- | --- | --- | --- |
| Goyen & Anshel, 1998 | 2 | 2 | 2 | 2 | 0 | 0 | 0 | 1 | 1 | 2 | 2 | 2 | 2 | 1 | **medium** |
| Reeves, Nicholls & McKenna, 2009 | 2 | 2 | 2 | 2 | 0 | 0 | 0 | 2 | 1 | 2 | N.A | 2 | 2 | 1 | **medium** |
| Crocker  & Graham, 1995 | 2 | 2 | 2 | 2 | 0 | 0 | 0 | 1 | 2 | 1 | 2 | 2 | 2 | 1 | **medium** |
| Thellwell, Weston & Greenlees, 2007 | 2 | 2 | 2 | 2 | 0 | 0 | 0 | 1 | 1 | 1 | N.A. | 2 | 2 | 1 | **medium** |
| Cumming, Smith, Grossbard, Smoll & Malina,2012 | 2 | 2 | 2 | 2 | 0 | 0 | 0 | 1 | 1 | 2 | 2 | 2 | 2 | 1 | **medium** |
| Nicholls, Levy, Carson., Thompson & Perry, 2016 | 2 | 2 | 2 | 2 | 0 | 0 | 0 | 1 | 1 | 2 | 0 | 2 | 2 | 1 | **medium** |

| **Publication** | **Question/objective described** | **Appropriate study design** | **Appropriate subject selection** | **Characteristics sufficiently described** | **Random allocation** | **Researchers blinded** | **Subjects blinded** | **Outcome measures well defined and robust to bias** | **Appropriate sample size** | **Analytic methods well described** | **Estimate of variance reported** | **Controlled for confounding** | **Results reported in detail** | **Conclusion supported by results?** | **Rating** |
| --- | --- | --- | --- | --- | --- | --- | --- | --- | --- | --- | --- | --- | --- | --- | --- |
| Krokosz & Jochaimek, 2018 | 2 | 2 | 2 | 2 | 0 | 0 | 0 | 1 | 1 | 2 | N.A. | 2 | 2 | 1 | **medium** |
| Deroche, Woodman, Stephan, Brewer & Le Scanff, 2011 | 2 | 2 | 2 | 2 | 0 | 0 | 0 | 2 | 1 | 2 | N.A | 2 | 2 | 0 | **medium** |
| Fogagca, 2021 | 2 | 2 | 2 | 2 | 0 | 0 | 0 | 1 | 1 | 1 | 2 | 2 | 2 | 1 | **medium** |
| Nicholls, 2007 | 1 | 2 | 2 | 2 | 0 | 0 | 0 | 1 | 0 | 1 | N.A. | 1 | 1 | 1 | **low** |
| Rutkowska, Bergier, & Witkowski, 2014 | 2 | 2 | 1 | 2 | 0 | 0 | 0 | 1 | 0 | 1 | N.A. | 1 | 1 | 1 | **low** |
| Sagar, Lavallee & Spray, 2009 | 2 | 2 | 1 | 2 | 0 | 0 | 0 | 1 | 1 | 1 | N.A. | 1 | 2 | 1 | **low** |
| Secades, Molinero, Salguero, Barquin, de la Vega & Marquez, 2016 | 2 | 2 | 2 | 2 | 0 | 0 | 0 | 1 | 2 | 2 | 2 | 1 | 2 | 1 | **medium** |

**Table 3** *continued: Qualsyst” quality assessment*

| **Publication** | **Question/objective described** | **Appropriate study design** | **Appropriate subject selection** | **Characteristics sufficiently described** | **Random allocation** | **Researchers blinded** | **Subjects blinded** | **Outcome measures well defined and robust to bias** | **Appropriate sample size** | **Analytic methods well described** | **Estimate of variance reported** | **Controlled for confounding** | **Results reported in detail** | **Conclusion supported by results?** | **Rating** |
| --- | --- | --- | --- | --- | --- | --- | --- | --- | --- | --- | --- | --- | --- | --- | --- |
| Bernacka, Sawicki, Mazurek-Kusiak, & Hawlena, 2016 | 2 | 2 | 2 | 2 | 2 | 0 | 0 | 1 | 1 | 2 | 1 | 2 | 1 | 1 | **medium** |
| McLoughlin, Fletcher, Slavich, Arnold & Moore, 2021 | 2 | 2 | 2 | 2 | 2 | 0 | 0 | 1 | 2 | 2 | 2 | 2 | 2 | 1 | **high** |
| Anshel, Sutasrso & Jubenville, 2009 | 2 | 2 | 2 | 2 | 0 | 0 | 0 | 1 | 2 | 2 | 2 | 2 | 2 | 1 | **medium** |
| Skein, Harrison & Clarke, 2019 | 2 | 2 | 2 | 2 | 0 | 0 | 0 | 1 | 0 | 2 | 2 | 2 | 2 | 1 | **medium** |
| Krisitiansen, & Roberts, 2010 | 2 | 2 | 2 | 2 | 0 | 0 | 0 | 1 | 0 | 2 | N.A. | 2 | 2 | 2 | **medium** |
| Litwick-Kaminska, 2020 | 2 | 2 | 2 | 2 | 0 | 0 | 0 | 1 | 1 | 2 | 2 | 2 | 2 | 1 | **medium** |
| Yi, Smith, & Vitiliano, 2005 | 2 | 2 | 2 | 2 | 0 | 0 | 0 | 1 | 1 | 2 | N.A. | 2 | 2 | 1 | **medium** |

**Table 3** *continued:* Qualsyst” quality assessment

**Table 3***: Qualsyst” quality assessment (continued)*

| **Publication** | **Question/objective described** | **Appropriate study design** | **Appropriate subject selection** | **Characteristics sufficiently described** | **Random allocation** | **Researchers blinded** | **Subjects blinded** | **Outcome measures well defined and robust to bias** | **Appropriate sample size** | **Analytic methods well described** | **Estimate of variance reported** | **Controlled for confounding** | **Results reported in detail** | **Conclusion supported by results?** | **Rating** |
| --- | --- | --- | --- | --- | --- | --- | --- | --- | --- | --- | --- | --- | --- | --- | --- |
| Szczypinska, Samelko & Guszkowska, 2021 | 2 | 2 | 2 | 2 | 1 | 0 | 0 | 1 | 1 | 2 | 1 | 2 | 2 | 2 | **medium** |
| Kerdijk, van der Kamp, & Polman, 2016 | 2 | 2 | 2 | 2 | 0 | 0 | 0 | 1 | 1 | 2 | N.A. | 2 | 2 | 1 | **medium** |
| Dolenc, 2015 | 2 | 2 | 2 | 2 | 2 | 0 | 0 | 1 | 1 | 2 | 2 | 2 | 2 | 2 | **high** |
| Pensgaard & Ursin, 1998 | 2 | 2 | 2 | 2 | 0 | 0 | 0 | 1 | 1 | 2 | 1 | 2 | 2 | 1 | **medium** |

N.A.: not applicable; 2 = yes; 1 = partial; 0 = no quality; quality score >= 75% high, 55-75% medium, <= 55% low

References

Leprince, C., D’Arripe-Longueville, F. & Doron, J. (2018). Coping in teams: Exploring athletes’ communal coping strategies to deal with shared stressors. *Frontiers in psychology*, 1908.

Guo, T., Ni, Y., Li, Q. & Hong, H. (2019). The Power of Faith: The Influence of Athletes’ Coping Self-Efficacy on the Cognitive Processing of Psychological Stress. *Frontiers in Psychology*, *10*, 1565.

Hill, A. P., Hall, H. K. & Appleton, P. R. (2010). Perfectionism and athlete burnout in junior elite athletes: The mediating role of coping tendencies. *Anxiety, Stress, & Coping*, *23*(4), 415-430.

Daumiller, M., Rinas, R. & Breithecker, J. (2021). Elite athletes’ achievement goals, burnout levels, psychosomatic stress symptoms, and coping strategies. *International Journal of Sport and Exercise Psychology*, 1-20.

Anshel, M., & Anderson, D. (2002). Coping with acute stress in sport: Linking athletes' coping style, coping strategies, affect, and motor performance. *Anxiety, Stress & Coping*, *15*(2), 193-209.

Britton, D. M., Kavanagh, E. J., & Polman, R. C. (2019). A path analysis of adolescent athletes’ perceived stress reactivity, competition appraisals, emotions, coping, and performance satisfaction. *Frontiers in Psychology*, *10*, 1151.

Raedeke, T. D. & Smith, A. L. (2004). Coping resources and athlete burnout: An examination of stress mediated and moderation hypotheses. *Journal of sport and exercise psychology*, *26*(4), 525-541.

Nicholls, A. R., Polman, R., Levy, A. R., Taylor, J. & Cobley, S. (2007). Stressors, coping, and coping effectiveness: Gender, type of sport, and skill differences. *Journal of sports sciences*, *25*(13), 1521-1530.

Goyen, M. J., & Anshel, M. H. (1998). Sources of acute competitive stress and use of coping strategies as a function of age and gender. *Journal of Applied Developmental Psychology*, *19*(3), 469-486.

Reeves, C. W., Nicholls, A. R. & McKenna, J. (2009). Stressors and coping strategies among early and middle adolescent premier league academy soccer players: Differences according to age. *Journal of Applied Sport Psychology*, *21*(1), 31-48.

Crocker, P. R. & Graham, T. R. (1995). Coping by competitive athletes with performance stress: Gender differences and relationships with affect. *The sport psychologist*, *9*(3), 325-338.

Thelwell, R. C., Weston, N. J. & Greenlees, I. A. (2007). Batting on a sticky wicket: Identifying sources of stress and associated coping strategies for professional cricket batsmen. *Psychology of Sport and Exercise*, *8*(2), 219-232.

Cumming, S. P., Smith, R. E., Grossbard, J. R., Smoll, F. L. & Malina, R. M. (2012). Body size, coping strategies, and mental health in adolescent female athletes. *International Journal of Sports Science & Coaching*, *7*(3), 515-526.

Nicholls, A. R., Levy, A. R., Carson, F., Thompson, M. A. & Perry, J. L. (2016). The applicability of self-regulation theories in sport: goal adjustment capacities, stress appraisals, coping, and well-being among athletes. *Psychology of Sport and Exercise*, *27*, 47-55.

Krokosz, D. & Jochimek, M. (2018). Coping strategies, perception of sport risk and satisfaction with life in men and women practicing extreme sports. *Balt J Health Phys Activ*, *10*(4), 238-245.

Deroche, T., Woodman, T., Stephan, Y., Brewer, B. W. & Le Scanff, C. (2011). Athletes' inclination to play through pain: a coping perspective. *Anxiety, Stress & Coping*, *24*(5), 579-587.

Fogaca, J. L. (2021). Combining mental health and performance interventions: Coping and social support for student-athletes. *Journal of Applied Sport Psychology*, *33*(1), 4-19.

Nicholls, A. R. (2007). A longitudinal phenomenological analysis of coping effectiveness among Scottish international adolescent golfers. European Journal of Sport Science, 7(3), 169-178.

Rutkowska, K., Bergier, J., & Witkowski, Z. (2014). Styles of coping with stress and locus of control in sporting situations in a group of young female football players. *Human Movement*, *15*(1), 60-64.

Sagar, S. S., Lavallee, D., & Spray, C. M. (2009). Coping with the effects of fear of failure: A preliminary investigation of young elite athletes. *Journal of Clinical Sport Psychology*, *3*(1), 73-98.

Sagar, S. S., Lavallee, D., & Spray, C. M. (2009). Coping with the effects of fear of failure: A preliminary investigation of young elite athletes. *Journal of Clinical Sport Psychology*, *3*(1), 73-98.

Bernacka, R. E., Sawicki, B., Mazurek-Kusiak, A. & Hawlena, J. (2016). Conforming and nonconforming personality and stress coping styles in combat athletes. *Journal of human kinetics*, *51*(1), 225-233.

McLoughlin, E., Fletcher, D., Slavich, G. M., Arnold, R. & Moore, L. J. (2021). Cumulative lifetime stress exposure, depression, anxiety, and well-being in elite athletes: A mixed-method study. *Psychology of sport and exercise*, *52*, 101823.

Anshel, M. H., Sutarso, T. & Jubenville, C. (2009). Racial and gender differences on sources of acute stress and coping style among competitive athletes. *The journal of social psychology*, *149*(2), 159-178.

Skein, M., Harrison, T. & Clarke, D. (2019). Sleep characteristics, sources of perceived stress and coping strategies in adolescent athletes. *Journal of sleep research*, *28*(4), e12791.

Kristiansen, E. & Roberts, G. C. (2010). Young elite athletes and social support: Coping with competitive and organizational stress in “Olympic” competition. *Scandinavian Journal of Medicine & Science in Sports*, *20*(4), 686-695.

Litwic-Kaminska, K. (2020). Types of cognitive appraisal and undertaken coping strategies during sport competitions. *International Journal of Environmental Research and Public Health*, *17*(18), 6522.

Yi, J. P., Smith, R. E., & Vitaliano, P. P. (2005). Stress-resilience, illness, and coping: a person-focused investigation of young women athletes. *Journal of behavioral medicine*, *28*(3), 257-265.

Szczypińska, M., Samełko, A. & Guszkowska, M. (2021). Strategies for coping with stress in athletes during the COVID-19 pandemic and their predictors. *Frontiers in Psychology*, 498.

Kerdijk, C., Van der Kamp, J. & Polman, R. (2016). The influence of the social environment context in stress and coping in sport. *Frontiers in psychology*, *7*, 875.

Dolenc, P. (2015). Anxiety, self-esteem and coping with stress in secondary school students in relation to involvement in organized sports. *Slovenian Journal of Public Health*, *54*(3), 222-229.

Pensgaard, A. M. & Ursin, H. (1998). Stress, control, and coping in elite athletes. *Scandinavian journal of medicine & science in sports*, *8*(3), 183-189.
